# Temporal distribution of gastroenteritis viruses in Ouagadougou, Burkina Faso: seasonality of rotavirus

**DOI:** 10.1186/s12889-017-4161-7

**Published:** 2017-03-21

**Authors:** Nafissatou Ouedraogo, Stephanie Moustapha Tomba Ngangas, Isidore Juste Ouindguèta Bonkoungou, Aissatou Bénéwendé Tiendrebeogo, Kuan Abdoulaye Traore, Idrissa Sanou, Alfred Sababénédjo Traore, Nicolas Barro

**Affiliations:** 1 Laboratoire de Biologie Moléculaire, d’Epidémiologie et de Surveillance des Bactéries et Virus Transmis par les Aliments, Centre de Recherche en Sciences Biologiques Alimentaires et Nutritionnelles (CRSBAN), Université Ouaga I Joseph KI-ZERBO, Ouagadougou, Burkina Faso; 2 Laboratoire National de Santé Publique, Direction de la Biologie médicale (DBM), Ouagadougou, Burkina Faso; 3 Laboratoire de Bactériologie-Virologie de l’Hôpital National BLAISE COMPAORE, Ouagadougou, Burkina Faso; 4 UFR Sciences de la Santé, Université Ouaga I Joseph KI ZERBO, Ouagadougou, Burkina Faso

**Keywords:** Virus, Gastroenteritis, Season, Climatic variables

## Abstract

**Background:**

Acute gastroenteritis is one of the most common diseases among children and adults, and continues to cause a major problem of public health in Burkina Faso. The temporal pattern of rotavirus, norovirus, sapovirus, astrovirus, adenovirus and *Aichivirus A* was studied by examining prevalence of gastroenteritis viruses in association with meteorological variables in Ouagadougou, Burkina Faso.

**Methods:**

Stool samples from 263 children under 5 years of age and 170 older children patients, adolescent and adults with gastroenteritis were collected in Ouagadougou, Burkina Faso from November 2011 to September 2012. Enteric viruses were detected using real-time or end-point (RT-) PCR. Temperature, humidity and monthly rainfall were recorded from the National Meteorological Direction. Categorical data were compared by Chi-square tests and the effect of weather variables and monthly prevalence were analyzed using Pearson Correlation Coefficient test.

**Results:**

The prevalence of rotavirus infections was significantly higher in the dry season (Season S1) compared to the wet season (season S2) (*p* = 0.03) among the population of children under 5 years of age. No statistically significant difference was observed regarding other gastroenteritis viruses comparing the dry season and the wet season. Positive cases of rotavirus, norovirus, adenovirus and sapovirus in children under 5 years of age were correlated with temperature (*r* = −0.68, *p* = 0.01; *r* = −0.74, *p* < 0.001; *r* = −0.68, *p* = 0.01; *r* = −0.65, *p* = 0.02, respectively) and only rotavirus, adenovirus and astrovirus were correlated with relative humidity (*r* = −0.61, *p* = 0.04; *r* = −0.54, *p* = 0.08; *r* = −0.51, *p* = 0.1 respectively). No correlation was observed with rainfall. In older children, adolescent and adults patients, rotavirus and norovirus correlated with relative humidity (*r* = −0.58, *p* = 0.05; *r* = 0.54, *p* = 0.08 respectively), but, no correlation was observed between the temperature and the rainfall.

**Conclusion:**

This study extends knowledge on the monthly fluctuations on the prevalence of viral gastroenteritis. These results can provide valuable information necessary to alert health care providers when a period of infection in the community is likely to occur. The transmission of these viruses in Burkina Faso could depends on multiple factors including climatic variables.

## Background

Acute gastroenteritis is one of the most common diseases among children and adults, and continues to be a major public health issue worldwide [1–5]. Viral gastroenteritis occurs with similar frequencies in developed and developing countries, but the seasonality differ from region to region [6, 7]. The epidemiology of infectious gastroenteritis is complex and multifactorial, involving both hosts and environmental factors [8, 9].

Seasonality of viral gastroenteritis has been extensively studied in temperate regions of the northern hemisphere [10, 11]. Indeed, local weather factors such as temperature, relative humidity, and rainfall have been suggested as important factors in the spread and seasonality of infectious gastroenteritis [9, 12, 13]. In contrast, very few data are available from Africa particularly in West Africa. The few existing data reported are ususally regardig rotavirus (RotaV, family *Reoviridae*) and norovirus (NoV, Family *Caliciviridae*) [14–17]. Thus, there is almost no data on the other viruses such as sapovirus (SaV, family *Caliciviridae*), human astroviruses (AstV, family *Astroviridae*), AdV, family *Adenoviridae*) and *Aichiviruses A* (AiV, family *Picornaviridae*) in Africa. These viruses are highly transmissible, primarily via the faecal-oral route, by ingestion water or contaminated food and also from person to person by airborne droplets [13].

In Burkina Faso, only the temporal distribution of rotavirus has been reported [18, 19]. However, the seasonality of other enteric viruses remains unknown. To our knowledge, this is the first report to describe the temporal pattern of infection of enteric viruses such as RotaV, NoV, SaV, AdV, AstV and AiV virus, and to analyse the association with weather variables. This information would be important for understanding the local epidemiology of enteric virus diarrhea and gain insights into what this seasonal pattern might mean for disease transmission.

## Methods

### Study area

**Fig. 1:**
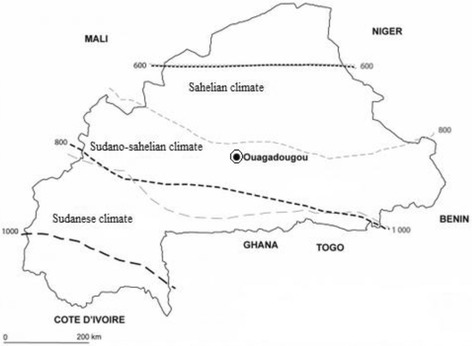
Map of Burkina Faso with climate areas (source National Meteorological Direction)

The study was conducted in Ouagadougou, the capital city of Burkina Faso. The city has a sudano-sahelian climate (Fig. 1). As in the rest of West Africa, two major seasons are recognized: A wet season from June to October and a dry season from November to May. The average rainfall is of the order 700 mm during the year and winds are marked by a dust. Three hospitals in Ouagadougou were selected for patients’ recruitment and the collection of stools samples: District hospital of Bogodogo, pediatric clinic “les Tissérins” and Medical center with surgical antenna “Paul VI”. These hospitals were the peripheral health centre.

### Participants and sampling

Four hundred thirty-third (433) patients (263 children under 5 years and 170 patients older children, adolescent and adults) with gastroenteritis were recruited at the three hospitals in Ouagadougou between November 2011 and September 2012. The study population was children aged less than 5 years (mean 14.1 months) and the older children, adolescents and adults aged 5–89 years (mean age of 30.7 years). All participants or guardians were informed on the study details and their oral consent was obtained and documented in the questionnaire form before stool specimen collection according to guidelines from the ministry of health. The study protocol and consent procedure was approved by the Ethics Committee of Burkina Faso and the ministry of health, 03 BP 7009. Ouagadougou 03, Burkina Faso. Stool samples were taken and stored at –20 ° C prior to analysis.

### Samples analyses by Real time PCR

Viral nucleic acids were extracted from 20% fecal suspensions in phosphate-buffered saline (PBS) using the NucliSENS^®^ EasyMAG™ platform (bioMérieux, Marcy l’Etoile, France).

All assays were run on an 7500 Real-Time PCR system (Applied Biosystems, Foster City, CA, USA) using the TaqMan^®^ Fast Virus One-Step Master Mix with primers and probes described previously for RotaV, NoV, SaV and AstV respectively [20]. AdV all types were detected using a commercial real-time PCR assay designed to detect all types of human AdV (Adenovirus R-gene™, Argène, Verniolle, France) and AiV were detected by end-point RT-PCR using the Qiagen One-Step RT-PCR kit (Qiagen, Hilden, Germany) and the primer set Ai6261/Ai6779 [20].

### Climatic variables

For the study period, monthly temperature (minimum and maximum), monthly humidity (minimum and maximum) and rainfall were recorded from the National Meteorological Direction (http://www.meteoburkina.bf). The mean values of climatic variables were calculated from daily values recorded. Seasons were defined from analysis of monitoring climatic variables: temperatures, relative humidity, wind and rainfall in the year. In this study we defined two major seasons according to the average values of daily and monthly temperatures, relative humidity, wind and rainfall: The dry season (S1) from November to May, followed to the wet season (S2) from June to October. The season S1 is subdivided into dry-cold period and dry-warm period.

### Statistical analysis

We compared categorical data by Chi-square tests. The level of confidence was 95% for all confidence intervals (CI). The relationship between monthly enteric virus prevalence and climatic variable was evaluated using parametric Pearson Correlation Coefficient test. Statistical analyses were performed with Stata® software (StataCorp release 10, 2007; College Station, TX, USA). A *p*-value less than 0.05 were considered statistically significant.

## Results

### Characteristics of seasons

Table 1 summarizes the average values of climate variables for each season. The season (S1) corresponds to the dry season, which extends from November to May. S1 is subdivided into cold period (mean temperature = 26.6 °C, November to February) and warm period (mean temperature = 32.4 °C, March to May). Wet Season (S2) is the rainy period which runs from June to October. A statistically significant difference was observed for humidity and rainfall values between S1 and S2 (*p* < 0.001*, p* < 0.001 respectively). No significant difference was observed for the temperature and wind speed between S1 and S2.

**Table 1:** Season characteristics during November 2011 to October 2012

Climatic variables	Seasons		
	Dry		Wet
	Cold	Hot	
Temperature (°C)^a^			
Mean Min	18.4	26.1	23.5
Mean max	34.7	38.6	33
Humidity (%)^b^			
Mean min	12.8	22.3	53.5
Mean max	44.8	55	90.3
Mean Rainfall (mm)^c^	0	29.16	227.6
Mean wind speed (m/s)^a^	2.4	3.2	2.5

^a^ *p* > 0.05,^b^ *p* < 0.001, ^c^ *p* < 0.001. The *p* value is calculated for the parameters dry vs wet

### Temporal pattern of gastroenteritis viruses detected

The temporal pattern of gastroenteritis viruses detected in this study is reported in Table 2. RotaV was detected significantly more during the dry season S1 (165/229; 72.1%; 95% CI [66.3–77.9%]) compared to the wet season, S2 (2/34; 5.9%; 95% CI [0–13.8%]; *p* < 0.05 for both seasons). In contrast, RotaV detection was lower in other age groups (older children, adolescents and adults) with seasonal prevalence ranging from 1.4% (1/71; 95% CI [0–4.1%]) to 12.1% (12/99; 95% CI [5.7–18.5%]) and there was not significant difference between S1 and S2. At the other gastroenteritis viruses, no significant differences was observed in the seasonal prevalence of NoV, SaV, AstV, AdV and AiV between dry season and wet season for all participant.

**Table 2:** Temporal pattern of gastroenteritis viruses detected

Participants	Season	N° of gastroenteritis cases	Prevalence of viral agent detected, *n* (%)					
			Rotavirus	Norovirus	Sapovirus	Astrovirus	Adenovirus	Aichi virus
Children less than 5 years	Dry^a^	229	165 (72.1%)	41 (17.9%)	25 (10.9%)	11 (4.8%)	70 (30.6%)	2 (0.9%)
	Wet^b^	34	2 (5.9%)	14 (41.2%)	2 (5.9%)	2 (5.9%)	12 (35.3%)	2 (5.9%)
	*p*		*0.03*	*0.18*	*0.09*	*0.1*	*0.05*	*0.27*
Older children, adolescent and adults	Dry^a^	99	12 (12.1%)	10 (10.1%)	4 (4%)	1 (1%)	39 (39.4%)	1 (1%)
	Wet^b^	71	1 (1.4%)	14 (19.7%)	7 (9.9%)	1 (1.4%)	23 (32.4%)	1 (1%)
	*p*		*0.05*	*0.12*	*0.13*	*0.36*	*0.47*	*0.36*

^a^Season S1, ^b^Season S2

### Seasonal fluctuations of gastroenteritis viruses

**Fig. 2:**
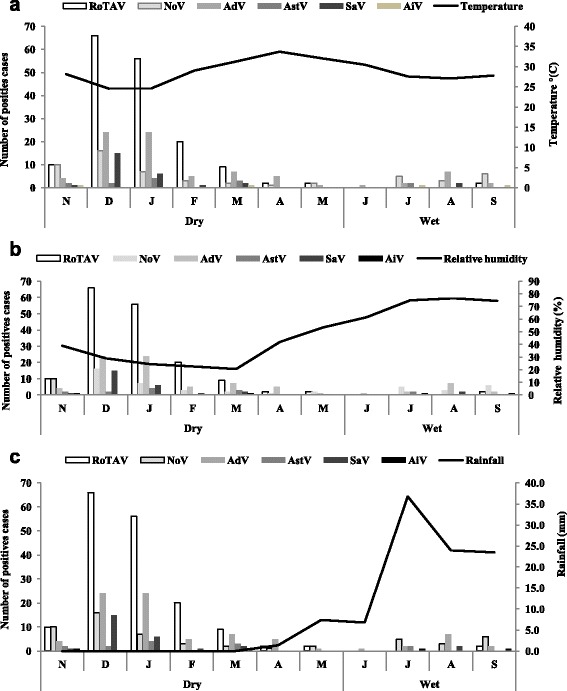
Variation of weather factors and samples positives for rotavirus, norovirus, adenovirus, astrovirus, sapovirus and aichi virus A in children less than 5 years in Ouagadougou, Burkina Faso from November 2011 to October 2012. **a** Temperature °(C), **b** Relative humidity (%) and **c** Rainfall (mm). Positives cases of rotavirus, norovirus, adenovirus and sapovirus correlated with temperature (*r* = −0.68, *r* = −0.74, *r* = −0.68, *r* = −0.65 respectively). Rotavirus, adenovirus and astrovirus correlated with relative humidity (*r* = −0.61, *r* = −0.54, *r* = −0.51 respectively). No correlation was observed with rainfall

**Fig. 3:**
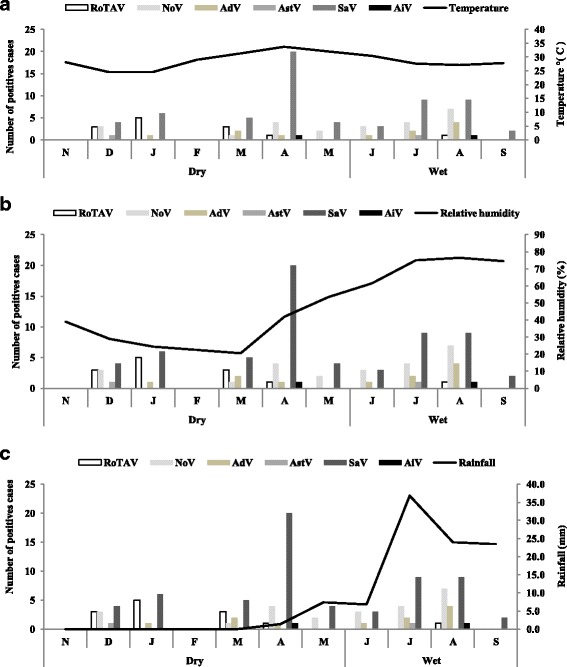
Variation of weather factors and samples positives for rotavirus, norovirus, adenovirus, astrovirus, sapovirus and aichi virus A in older children, adolescents and adults in Ouagadougou, Burkina Faso from November 2011 to October 2012. **a** Temperature °(C), **b** Relative humidity (%) and **c** Rainfall (mm). Positives cases of rotavirus and norovirus correlated with relative humidity (*r* = −0.58, *r* = 0.54 respectively). No correlation was observed with temperature and rainfall

The seasonal fluctuations of gastroenteritis viruses are shown in Figs. 2 and 3. In children less than 5 years, at least one viral gastroenteritis agent circulated throughout the study period with low activity in May and June. RotaV peak was observed from November to February, corresponding to the dry and cold period with low temperature, low relative humidity and little rainfall. Few rotavirus infections were detected in the other months of the study period. Even positives cases repartition were observed for other viral gastroenteritis agent, with very low peak in the dry and cold season (Fig. 2). In effect, NoV was identified throughout the year with 65.5% (36/55; 95% CI [52.9%-78.1%]) of the positive cases detected between November and February corresponding to the cold period of the dry season (Fig. 2). The majority of SaV was detected in the cold period of the dry season with 85.2% (23/27; 95% CI [71.8–98.6%]) of positives cases with no case observed between April to July and in September. Positive cases of adenoviruses were found throughout the year with 58.5% (48/82; 95% CI [47.8–69.2%]) of cases in December and January. No cases of AstV were observed between April to June and August to September.

Positives cases of RotaV, NoV, AdV and SaV correlated with temperature (*r* = −0.68, *p* = 0.01; *r* = −0.74, *p* < 0.001; *r* = −0.68, *p* = 0.01; *r* = −0.65, *p* = 0.02, respectively). RotaV, AdV and AstV correlated with relative humidity (*r* = −0.61, *p* = 0.04; *r* = −0.54, *p* = 0.08; *r* = −0.51, *p* = 0.1 respectively). No correlation was observed with rainfall.

In older children, adolescent and adults, at least one viral gastroenteritis agent was observed throughout the study period (Fig. 3). In contrast to children less than 5 years of age, no peaks were observed in the dry and cold period and the infection repartition for each virus was similar throughout the study period. RotaV was found in 61.5% (8/13; 95% CI [35–88%] of positives cases in the cold period of the dry season and no cases were observed in February, March to July, and September. NoV was detected throughout the year, except January, February and September, with 58.3% (14/24; 95% CI [38.6–78%]) of cases found in the wet season. The majority of AdV was identified in June to August corresponding to the wet season with 63.3% (7/11; 95% CI [34.8–91.8%]) of cases. AstV was detected only in December and July and AiV was detected only in April and August. However, high number of SaV infections was observed in April, June and August. Positives cases of RotaV and NoV correlated with relative humidity humidity (*r* = −0.58, *p* = 0.05; *r* = 0.54, *p* = 0.08 respectively). No correlation was observed with temperature and rainfall.

## Discussion

In this study we observed seasonal fluctuations in the prevalence of gastroenteritis virus (AdV RotaV, NoV, SaV, AstV and AiV). Cases of viral gastroenteritis in our study population were significantly associated with climatic variables (temperature and/or relative humidity).

During the whole period of surveillance, RotaV exhibited strong seasonality in children under 5 years, with predominance in occurred in the dry and relatively cold season (*p* < 0.03). These results are similar to those reports in children under 5 years in some countries in Africa (Burkina Faso, northern Ghana, Tunisia, Congo, and Guinea-Bissau) [14, 18, 19, 21–23]. Other researchers in Africa reported [24] that rotavirus infections were present throughout the year with a high prevalence in certain time of the year in Africa).

The seasonal peak of RotaV found in this study correlated with temperature and relative humidity. In Europe, RotaV gastroenteritis peaks were observed in late winter or early spring suggesting a role of meteorological factors in transmission of the virus [10, 11, 25, 26]. Observational studies of human rotavirus disease have suggested that the low temperature, lower humidity and lower precipitation levels is associated with an increased risk of rotavirus infection and could create favorable conditions for the RotaV spread, transmission and maintenance in environment [17, 27]. Indeed, cold weather causes people to stay in more closed areas and sensitive people can be in contact with surfaces or objects contaminated with a higher frequency and intensity. Similarly, it is known that respiratory viruses infections are spread by droplet contact in confined spaces and in circumstances more cluttered life [28].

However, in older children, adolescents and adults through there were a lack of seasonality of RotaV, which could be explained by their low susceptibility to rotavirus infection and which usually is asymptomatic [2].

Monitoring of NoV in our study population showed a relatively high prevalence during the wet season although it was not statistically significant (Table 2). This finding was similar to that Ayukenbong et al. [15] in Cameroon who reported that NoV circulated throughout the year with major peak in the beginning of the wet season between June and August. Furthermore rainfall has been highlighted as an important factor in seasonality for norovirus probably due to waterborne transmission of the virus [29–32]. Similarly, cool temperatures, low immunity of the population and the emergence of new variants are associated with increased activity of NoV [33]. Indeed, studies have shown that pediatric cases of NoV predominately are observed in winter with a peak occurring in November and December [13, 34]. Climate change has thus the potential to change the seasonality of NoV [35].

Regarding other gastroenteritis virus (AdV, AstV, SaV and AiV) we observed a relative similar distribution throughout the study period with some weak seasonal peaks in certain periods of the year. This seasonal distribution was not statistically significant. A similar pattern has been observed in other studies [36–38]. These results could be explained by a complex and multifactorial pattern involving both transmission to and between hosts and environmental factors.

## Conclusion

RotaV infections are more common during the cold and dry season and precipitation could increase the transmission of norovirus. Other gastroenteritis viruses were distributed similarly throughout the year. The limit of this study was that the study period of 1 year does not allow testing the consistency of seasonal trends of these viruses, which may change from 1 year to another. Despite this limit, this study extends knowledge on the monthly fluctuations on the prevalence of viral gastroenteritis. The transmission of these viruses could be multifactorial including climate variables. This data can provide valuable information necessary to alert health care providers when a period of infection in the community is likely to occur. Further studies with a longer period covering larger areas in Burkina Faso are required to test the consistency of this seasonal trend.

## Abbreviations

AdV: Adenovirus
AiV: Aichi virus A
AstV: Astrovirus
CI: Confidence intervals
NoV: Norovirus
RotaV: Rotavirus
SaV: Sapovirus
